# Optical coherence tomography angiography features of macular epiretinal neovascularization in Eales’ disease: a case report

**DOI:** 10.1186/s12348-022-00289-z

**Published:** 2022-05-06

**Authors:** Nora A. Alyousif, Abrar K. Alsalamah, Sawsan R. Nowilaty, Hassan A. Al-Dhibi

**Affiliations:** Vitreoretinal Division, King Khaled Eye Specialist Hospital, Riyadh, Saudi Arabia

## To the editor

Herein we report a case which highlights the optical coherence tomography angiography (OCTA) and multimodal retinal imaging features of macular epiretinal neovascularization (ERN) in Eales disease.

## Introduction

Eales disease (presumed tuberculous retinal vasculitis) is characterized by retinal vasculitis, vascular occlusions, peripheral retinal neovascularization, with or without optic nerve head neovascularization, and recurrent episodes of vitreous hemorrhage [1]. The condition is associated with mycobacterium tuberculosis, but its diagnosis requires excluding other causes of retinal neovascularization or inflammation which share similar features [2]. Although Eales disease primarily affects the peripheral retina, posterior involvement, namely macular edema or epimacular membranes, is seen. Macular ERN is, however, uncommon. This report describes a case of macular ERN presenting in Eales disease in which multimodal retinal imaging including OCTA permitted to demonstrate the ERN’s origin, features, and evolution following panretinal photocoagulation.

## Case report

**Fig. 1:**
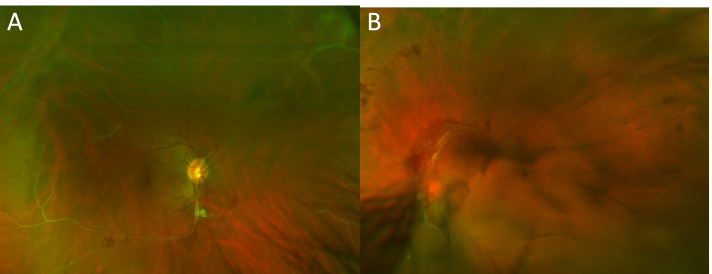
Fundus photograph of the right eye at presentation (**A**) showing peripheral perivenous sheathing, fibrous proliferation along the superior and inferior vascular arcades, neovascularization of the optic disc, and macular ERN. The view of the left fundus is obscured by vitreous hemorrhage but posterior fibrovascular proliferation and peripheral sclerosed vessels are seen (**B**)

**Fig. 2:**
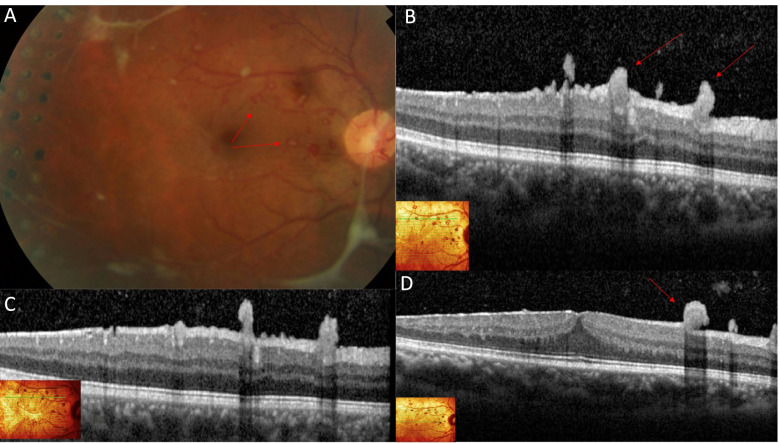
Colour fundus image (**A**) of the right eye showing multiple tiny vascular tufts on the macula (Red arrows) representing ERN. Right macular OCT at different cuts (**B**, **C**, **D**) shows the ERN as multiple hyperreflective lesions breaching the ILM and extending towards the vitreous (Red arrows)

**Fig. 3:**
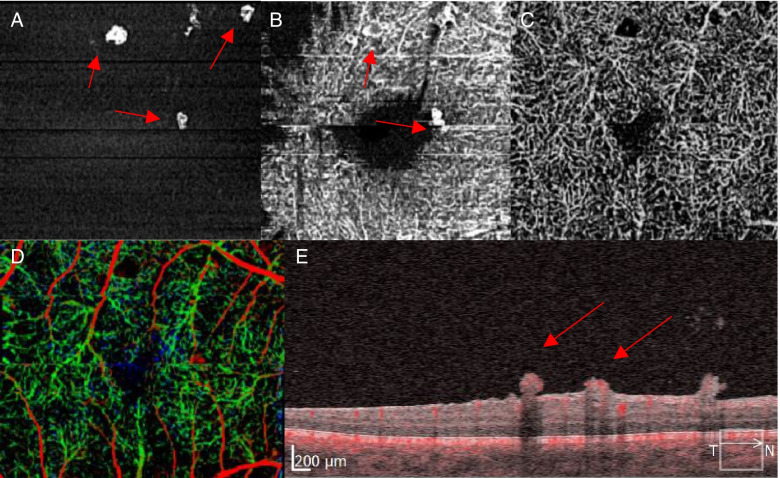
OCTA of the right macula showing the tiny epiretinal neovascular tufts extending towards the vitreous (**A**, **B**) (arrows). These ERN arise from the superficial retinal capillary plexuses, as seen on the superficial retinal slab then disappear when moving down to the deep retinal slab (**C**). The depth-coded view and corresponding B-scan show flow signal through the ERNs, and the signal extends above the ILM (arrows) (**D**, **E**)

This report was approved by the Institutional Review Board (IRB). A 35-year-old man with no known systemic disorders presented with a four-day history of floaters and decreased vision in the left eye. On ophthalmic examination, the best-corrected Snellen visual acuity was 20/20 in the right eye and 20/300 in the left eye. Intraocular pressure and anterior segments were normal in both eyes. Fundus examination of the right eye showed sclerosed retinal blood vessels, neovascularization of the optic disc (NVD) and elsewhere (NVE) with fibrovascular extensions towards the inferior vascular arcade, and tiny neovascular tufts on the macular surface (Fig. 1A). The left fundus view was mostly obscured by vitreous hemorrhage but posterior fibrovascular proliferation and peripheral sclerosed vessels were detected (Fig. 1B). Fluorescein angiography (FA) (Optos PLC, Dunfermline, UK) of the right eye showed widespread peripheral retinal capillary nonperfusion and leakage of dye from the NVD and the epimacular vascular tufts seen on funduscopy.. Macular Spectral-domain optical coherence tomography (SD-OCT) (Spectralis OCT, Heidelberg Engineering, Inc., Heidelberg, Germany) of the right eye depicted an epiretinal membrane and showed the vascular tufts as compact epiretinal hyper-reflective lesions, breaching the internal limiting membrane (ILM) towards the vitreous (Fig. 2). OCTA of the right macula confirmed that these epimacular tufts represent ERN by demonstrating intrinsic flow signal which extended beyond the ILM towards the vitreous (Fig. 3E). Moreover, these macular ERNs appeared to stem from the superficial retinal capillary plexuses (Fig. 3B).

**Fig. 4:**
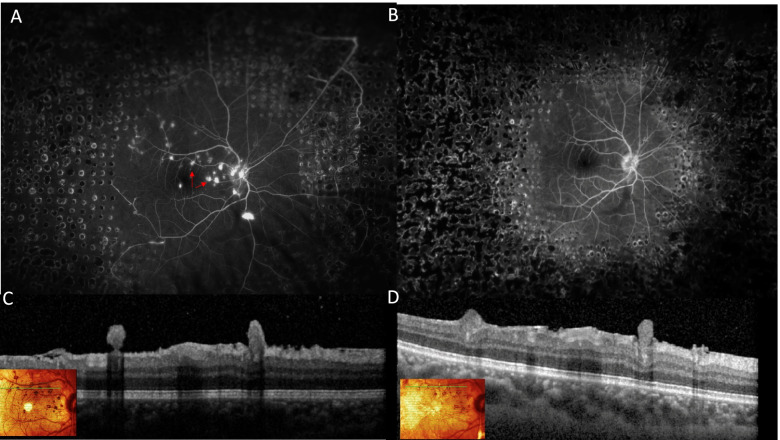
Ultra-widefield late-frame FA of the right eye (**A**) showing scatter laser photocoagulation marks to the nonperfused retinal zones with regression of NVD and NVE. Note the persistence of ERNs (red arrows). Following more extensive PRP, fluorescein leakage from the ERNs halted (**B**). Corresponding OCT (**D**) showing shrinkage in lesions’ size as compared to (**C**)

**Fig. 5:**
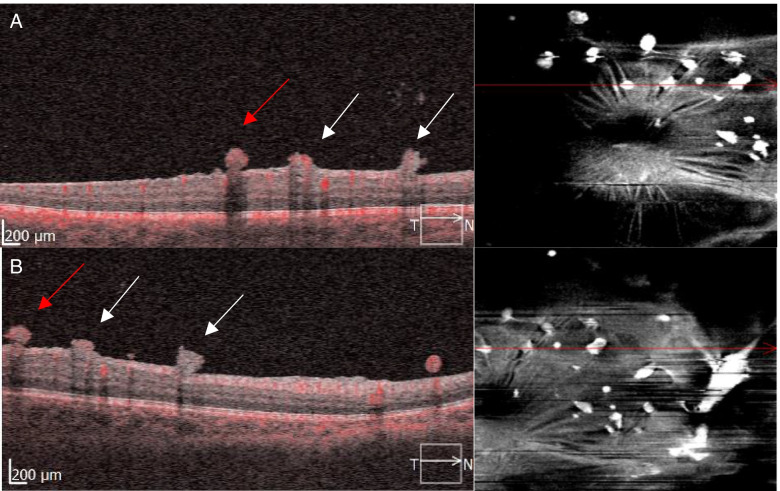
OCTA at the time of initial treatment shows intrinsic flow signal through these macular ERN (**A**). After the last session of laser ablation, decreased flow signal was observed in some ERN lesions (white arrows) but flow persisted in others (red arrow) (**B**)

Laboratory investigations including complete blood cell count, fasting blood sugar, sickle cell screen, thrombophilia screen, antinuclear antibody, antineutrophil cytoplasmic antibody serum angiotensin-converting enzyme, lysozyme, syphilis serology, and QuantiFERON-TB Gold were within normal limits. Chest radiograph was unremarkable. Tuberculin skin test was positive (15 × 15 mm induration). With this constellation of findings, the diagnosis of Eales disease was made, and the patient was started on empiric anti-tubercular therapy consisting of isoniazid, rifampin, and pyrazinamide, to which oral corticosteroids at a dose of 1 mg/kg body weight were added 3 days later then tapered slowly. At 2 months, anti-tubercular therapy was reduced to isoniazid and rifampin for an additional 6 months. Concomitantly, several sessions of scatter argon laser photocoagulation were applied to both eyes. In the right eye, the laser was initially applied to the nonperfused retinal zones guided by FA. This allowed regression of the NVD and NVE. However, the macular ERN persisted, continuing to exhibit leakage on FA (Fig. 4A). Following more extensive panretinal laser photocoagulation (PRP), fluorescein leakage from the ERNs halted, but some intrinsic flow signal persisted on OCTA suggesting incomplete regression (Figs. 4B and 5). Observation was elected. At 6 months, the right fundus appearance was stable. In the left eye, the vitreous hemorrhage had decreased following PRP with visual acuity returning to 20/25.

## Discussion

Macular ERN has been described in diabetic retinopathy, retinal venous occlusions, macular telangiectasia type 2 and retinitis pigmentosa [3–5]. However, to our knowledge, macular ERN has not been reported in Eales disease. OCTA is a valuable modality that identifies the vascular structures and their location within or beneath the retina and/or retinal pigment epithelium [6]. K.Rajurkar et al. reviewed the OCTA features of common macular changes in Eales disease, but macular ERN was not observed [7]. In our case OCTA confirmed the presence of ERN and showed that the epimacular ERN arose from the superficial vascular plexus.

It is known that the ERN in inflammatory conditions may regress with control of the inflammation [8]. However, faced with significant retinal non-perfusion, retinal ablation is usually necessary. In our case, the large NVD and NVE showed adequate regression after targeted ablation of the areas of retinal capillary non-perfusion. However, the macular ERNs failed to initially respond in to this treatment, demonstrating persistent reddish tufts on the macular surface with continued dye leakage on FA. This may suggest that macular ERN is a correlate of more extensive vaso-endothelial growth factor (VEGF) expression that cannot be appreciated by clinical examination or FA, or alternatively, that ERN arises from specific alterations of the macular microvascular structures that are not yet fully understood. Inflammation may have also triggered or contributed to the growth of the ERN. However, because in our case systemic anti-inflammatory and microbial therapy were concomitantly administered with retinal ablation, the exact trigger and optimal treatment modality of ERN in the context of Eales disease, or other occlusive retinal vasculitis, remain to be identified. Furthermore, the response of macular ERN to intravitreal anti-VEGF agents has been reported [5]. Anti-VEGF agents may provide further regression of the ERN. However, in our case, in view of the good regression of the major neovessels as well as the delayed, yet relative regression of the macular ERN and the 20/20 visual acuity in the right eye, additional anti-VEGF therapy was not elected. Further studies should shed more light in the exact role, if any, of anti-VEGF therapy for macular ERN.

## Data Availability

Not applicable.

## Abbreviations

ERN: Epiretinal neovascularization
FA: Fluorescein angiography
SD-OCT: Spectral domain optical coherence tomography
OCTA: Optical coherence tomography angiography
ILM: Nternal limiting membrane
NVD: Neovascularization of the optic disc
NVE: Neovascularization elsewhere
